# Supplementary material for: Cell softness renders cytotoxic T lymphocytes and T leukemic cells resistant to perforin-mediated killing
Source: Nat Commun. 2024 Feb 15;15:1405. doi: 10.1038/s41467-024-45750-w (PMC10869718; doi:10.1038/s41467-024-45750-w)
Supplement: Supplementary file 1 — Supplementary Information [file 41467_2024_45750_MOESM1_ESM.pdf]

Supplementary Information

Cell softness renders CTLs and T-leukemic cells resistant to perforin-mediated killing

Supplemental Fig. 1 Mechanical softness mediates the resistance of CD8^{+} effector T cells perforin-induced pore formation.

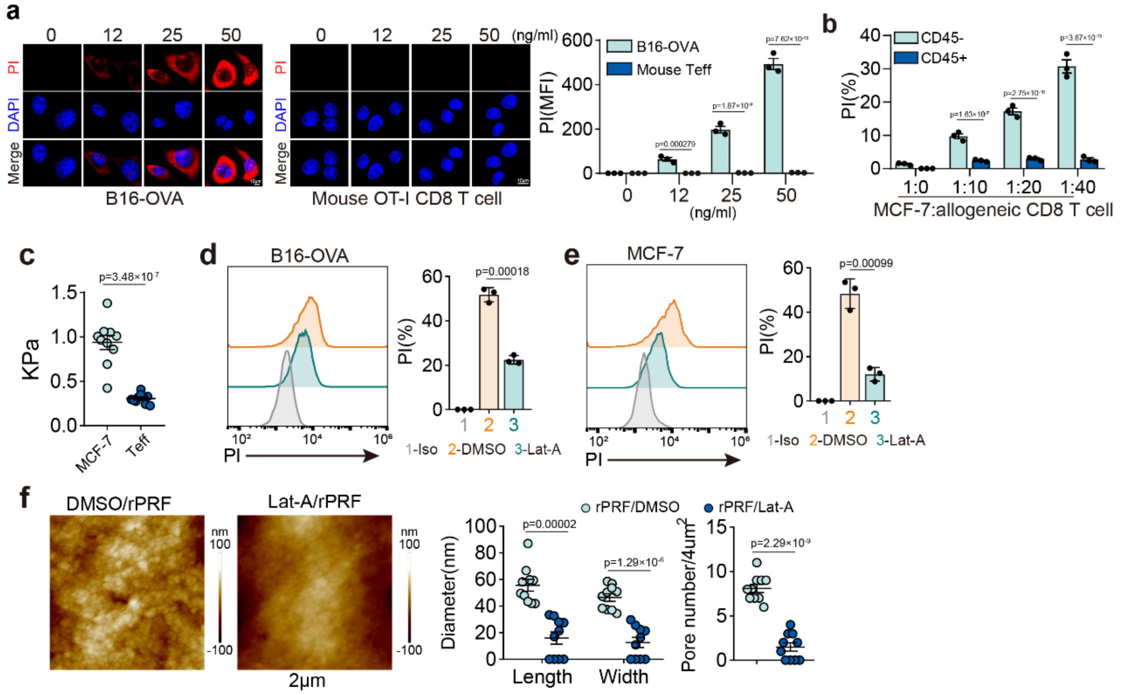

a OVA-B16 cells or OT-I CTLs were treated with recombinant perforin for 10 min, and PI was added to the medium. PI^{+} cells were analyzed by confocal microscopy. Scale bar, 10 μm. the MFI of PI were analyzed by Image J. **b** CD45^{-} MCF-7 cells were co-cultured with CD45^{+} Human CD8^{+} CTLs at different ratios for 4 hr. PI^{+} cells were analyzed by flow cytometry. **c** The stiffness of MCF-7 or human CD8^{+} effector T cells were determined by AFM. **d**, **e** B16 (**d**) or MCF-7 (**e**) were pretreated with 250nM Lat-A for 12 hr and then treated with Perforin for 10 min. The PI^{+} cells were analyzed by flow cytometry. **f** Human CD8^{+} Tn cells were pretreated with 250nM Lat-A for 12 hr and then treated with Perforin for 5 min. The cells were analyzed by AFM. The formed pore size and numbers were calculated. PI, Propidium lodide; Lat-A, latrunculin A; MFI, mean fluorescence intensity; PFR, perforin; AFM, atomic force microscopy; CTLs, cytotoxic T lymphocytes. n=3 independent experiments (a, b, d, e); n=10 independent experiments (c, f). The data are represented as mean ± SD. *p* value by one-way ANOVA Bonferroni's test (a,b and d-f); *p* value by two-tailed Student's t-test (c,f). Source data are provided as a Source Data file.

Supplemental Fig. 2 CTL was resistant to perforin pore formation by Filamin A.

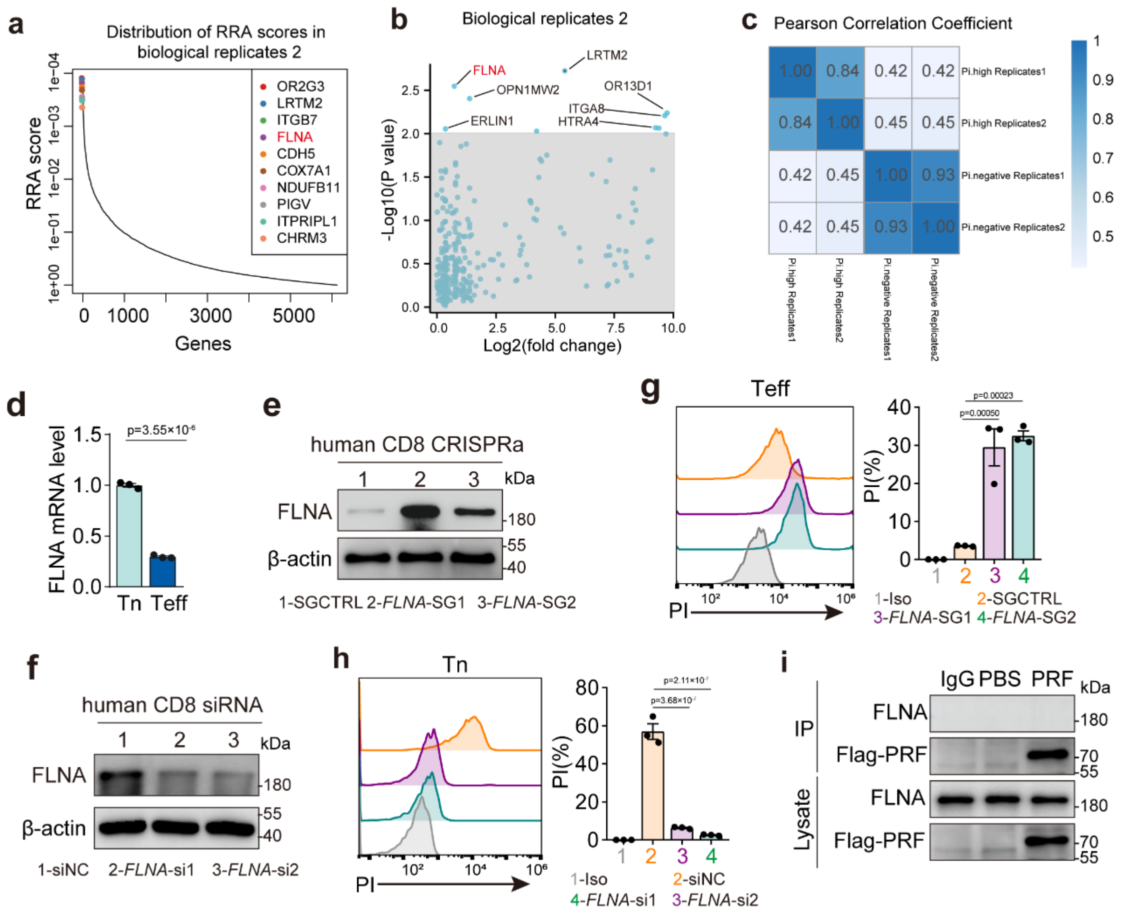

a Genes significantly enriched after Perforin treatment were identified through analysis of sequencing results in the MAGeCK program**.** **b** Significant hits from screens in cells treated with Perforin. line indicates Bonferroni-corrected significance threshold. **c** Correlation of sgRNA sequencing read counts in two biologic replicates. **d** The expression of *FLNA* in mouse CD8$^+$ Tn and Teff cells were analyzed by real-time PCR. **e** The expression of FLNA in human CD8$^+$ Teff cells transfected with or without *FLNA* overexpression by CRISPRa was determined by western blot. **f** The expression of FLNA in human CD8$^+$ Tn cells transfected with or without *FLNA* siRNA was determined by western blot. **g** The same as **e**, except that the cells were treated with Perforin for 10 min and then the PI$^+$ cells were analyzed by flow cytometry. **h** The same as **f**, except that the cells were treated with Perforin for 10 min and then the PI$^+$ cells were analyzed by flow cytometry. **i** Human CD8$^+$ Teff cells were treated with Perforin for 10 min. The cell lysate was extracted to perform an immunoprecipitation (IP) assay with anti-Flag-perforin antibody. The expression of FLNA was analyzed by western blot. Tn, naïve T cell; Teff, effector T cell; FLNA, Filamin A; n=3 independent experiments (d-i). The data are represented as mean ± SD. *p* value by one-way ANOVA Bonferroni's test (g, h); *p* value by two-tailed Student's t-test (d). Source data are provided as a Source Data file.

Supplemental Fig. 3 YAP negatively mediates FLNA expression in activated CTLs.

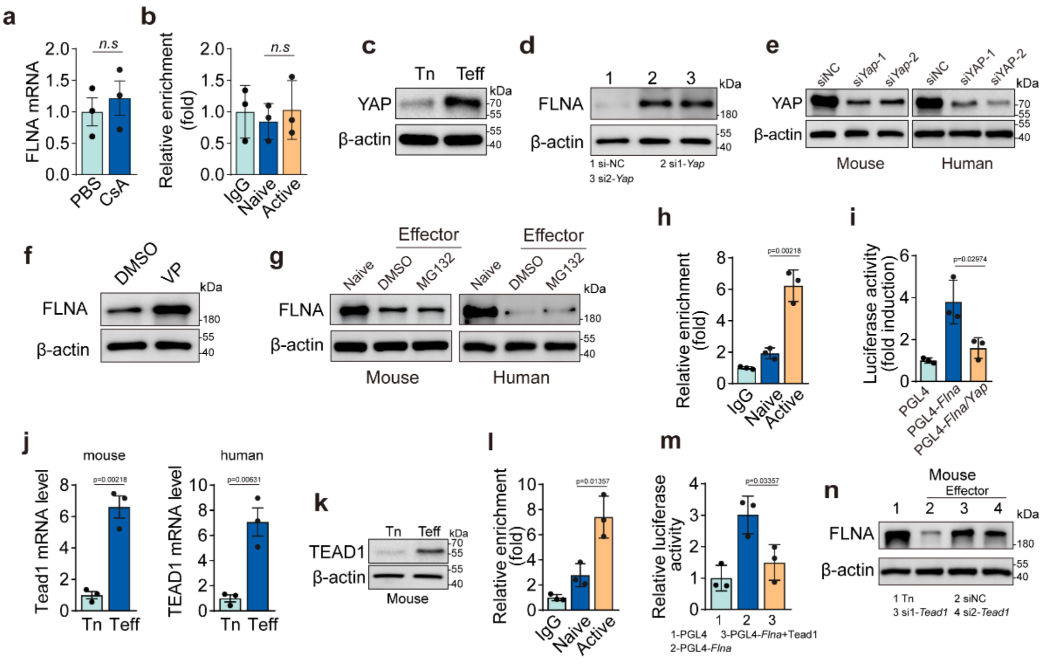

a The expression of *FLNA* in mouse CD8$^+$ Teff cells treated with or without CsA(1μM) were analyzed by real-time PCR. **b** ChIP–qPCR analysis of NFATc1 enrichment around the promoters of *FLNA* in mouse CD8$^+$ Tn and Teff cells. **c** The expression of YAP in mouse CD8$^+$ Tn and Teff cells were analyzed by western blot. **d** Mouse CD8$^+$ Teff cells were transfected with or without *YAP* siRNA, and FLNA expression was determined by western blot. **e** The expression of YAP in human or mouse CD8$^+$ Teff cells transfected with or without *YAP* siRNA was determined by western blot. **f** Mouse CD8$^+$ Teff cells were treated with DMSO or verteporfin (1μM) for 24 hr, and FLNA expression was determined by western blot. **g** Human or mouse CD8$^+$ Tn and Teff cells were treated with or without MG132 (1μM) for 4 hr, and FLNA expression was determined by western blot. **h** ChIP–qPCR analysis of YAP enrichment around the promoters of *FLNA* in mouse CD8$^+$ Tn and Teff cells. **i** NIH3T3 cells were co-transfected with *Flna* promoter-luciferase reporter PGL4 and Yap plasmid for 24 hr, followed by analysis of luciferase activity. **j** The expression of *FLNA* in human or mouse CD8$^+$ Tn and Teff cells were analyzed by real-time PCR. **k** The expression of TEAD1 in mouse CD8$^+$ Tn and Teff cells were analyzed by western blot. **l** ChIP–qPCR analysis of Tead1 enrichment around the promoters of *FLNA* in mouse CD8$^+$ Tn and Teff cells. **m** NIH3T3 cells were co-transfected with *Flna* promoter-luciferase reporter PGL4 and *Tead1* plasmid for 24 hr, followed by analysis of luciferase activity. **n** The expression of FLNA in mouse CD8$^+$ Tn cells or Teff cells transfected with or without *Tead1* siRNA was determined by western blot. CsA, cyclosporine A; YAP, yes associated protein; VP, verteporfin. n=3 independent experiments (a-n). The data are represented as mean ± SD. *p* value by one-way ANOVA Bonferroni's test (b, h-i, l-m); *p* value by two-tailed Student's t-test (a, j). Source data are provided as a Source Data file.

Supplemental Fig. 4 FLNA regulated the softness of T leukemic cells by YAP.

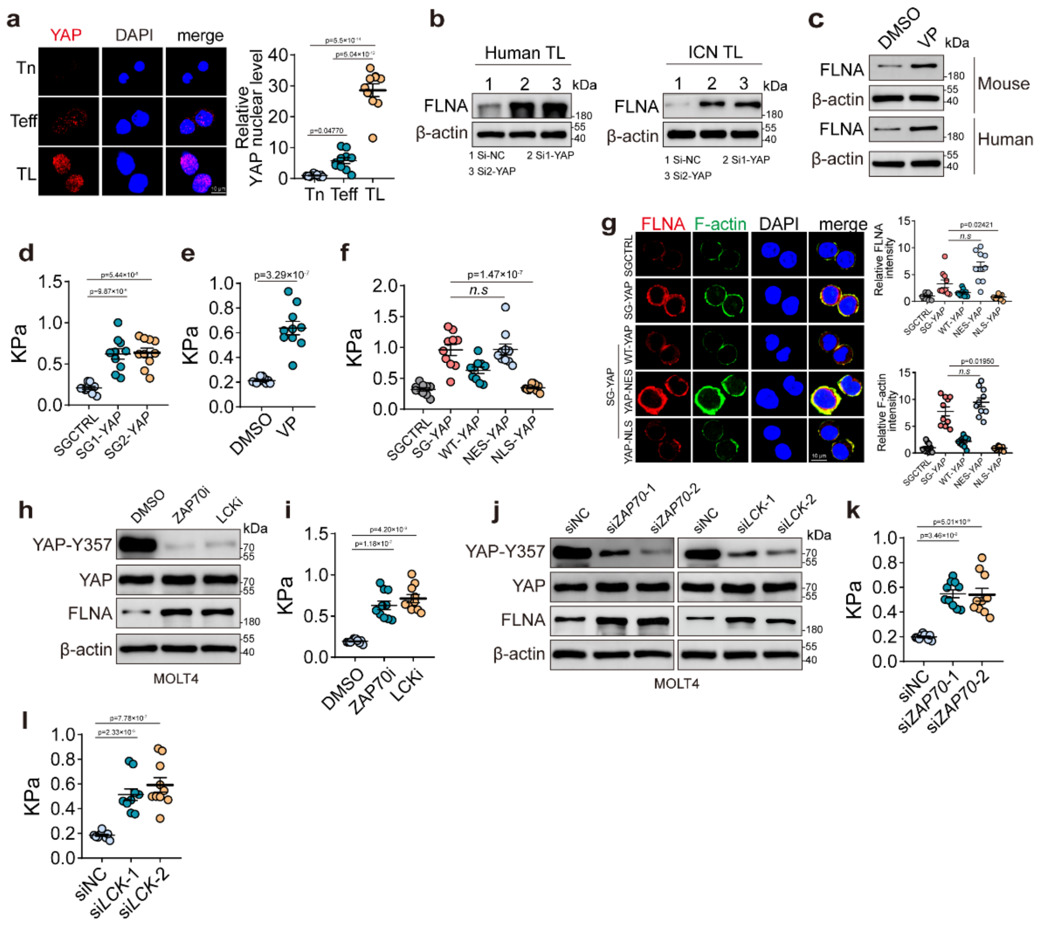

a Human CD8+ T naïve (Tn) and effector (Teff) and T leukemic cells (TL) were stained with DAPI (blue), YAP (red). Scale bar, 10 μm. **b** Human or mouse T leukemic cells were transfected with or without *YAP* siRNA, and FLNA expression was determined by western blot. **c** Human or mouse T leukemic cells were treated with DMSO or verteporfin (1μM) for 24 hr, and FLNA expression was determined by western blot. **d** Stiffness of Molt4 cells transfected with or without *YAP* sgRNA were determined by AFM. **e** Stiffness of human T leukemic cells treated with DMSO or verteporfin (1μM) for 24 hr were determined by AFM. **f** Stiffness of SGCTRL, YAP-SG, YAP-SG/NLS-YAP, YAP-SG/NES-YAP or YAP-SG/WT-YAP-Molt4 cells was analyzed by AFM (**f**). **g** the same as **f**, except that the cells were stained with DAPI (blue), phalloidin (F-actin, green) and FLNA (red). Scale bar, 10 μm. **h**, **i** The expression of FLNA, YAP and Y357-YAP in Molt4 treated with or without ZAP70 or LCK inhibitor were analyzed by western blot (**h**). Cell stiffness was determined by AFM(**i**). **j-l** The expression of FLNA, YAP and Y357-YAP in Molt4 transfected with or without ZAP70 or LCK siRNA were analyzed by western blot (**j**). Stiffness of cells transfected with or without *ZAP70*(**k**) or *LCK*(**l**) siRNA were determined by AFM. n=10 independent experiments (a, d-g, i, k, l); n=3 independent experiments (b, c, h, j). The data are represented as mean ± SD. *p* value by one-way ANOVA Bonferroni's test (a, d, f, g, i, k and l); *p* value by two-tailed Student's t-test (e). Source data are provided as a Source Data file.

Supplemental Fig. 5. T-leukemic cells softness resist perforin pore formation by YAP.

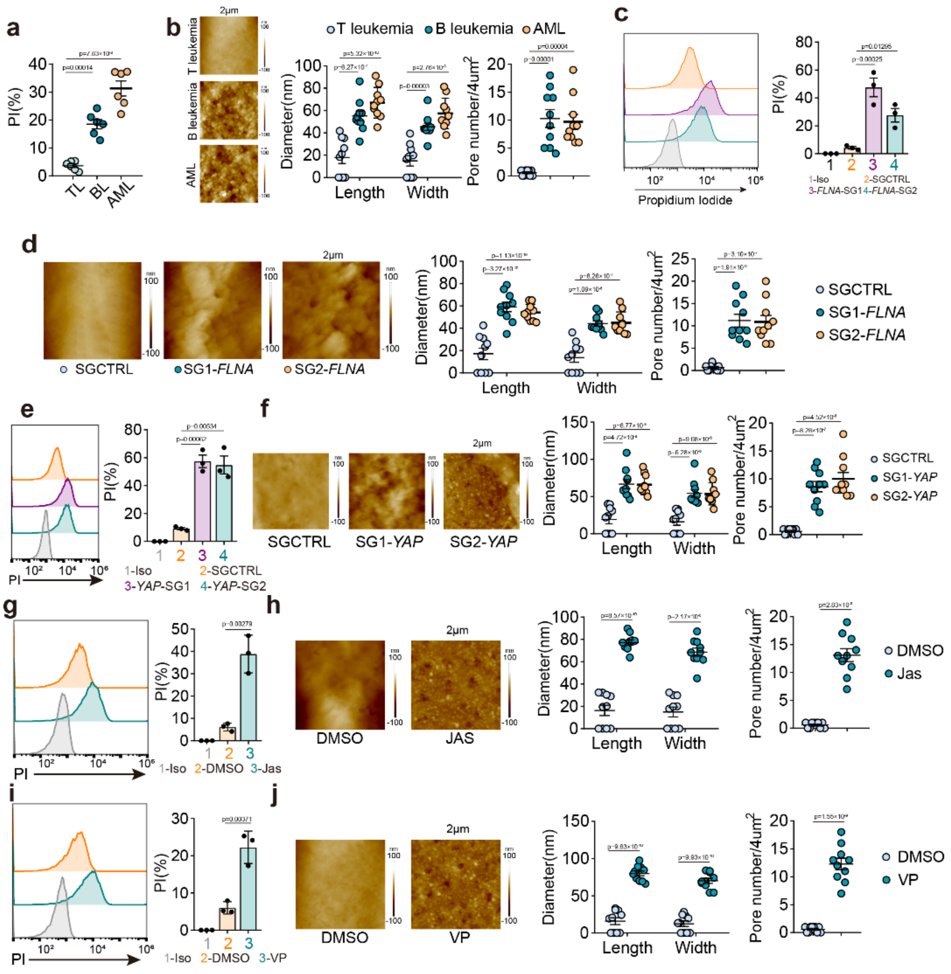

a, **b** Primary T leukemia (TL), B leukemia (BL) and AML were treated with Perforin for 10 min. The PI+ cells were analyzed by flow cytometry (**a**) and then imaged by AFM. The formed pore size and number were calculated (**b**). **c, d** Primary T leukemic cells transfected with or without FLNA overexpression were treated with Perforin for 10 min. The PI+ cells were analyzed by flow cytometry (**c**). The formed pore size and number were determined and calculated by AFM. (**d**). **e**, **f** Primary T leukemic cells with or without *YAP* sgRNA were treated with Perforin for 10 min. The PI+ cells were analyzed by flow cytometry (**e**). The formed pore size and number were determined and calculated by AFM. (**f**). **g-j** Primary T leukemic cells were pretreated with DMSO or 200 nM Jas or 1μM VP for 12 hr and then treated with Perforin for 10 min. The PI+ cells were analyzed by flow cytometry (**g** and **i**). The formed pore size and number were determined and calculated by AFM. (h and j). Jas, Jasplakinolide; AML, Acute myelogenous leukemia. n=6 independent experiments (a); n=10 independent experiments (b, d, f, h, j); n=3 independent experiments (c, e, g, i). The data are represented as mean ± SD. *p* value by one-way ANOVA Bonferroni's test (a-j); *p* value by Log-rank survival analysis (h, j). Source data are provided as a Source Data file.

Supplemental Fig. 6. T-leukemic cell softness regulates perforin pore formation in vivo.

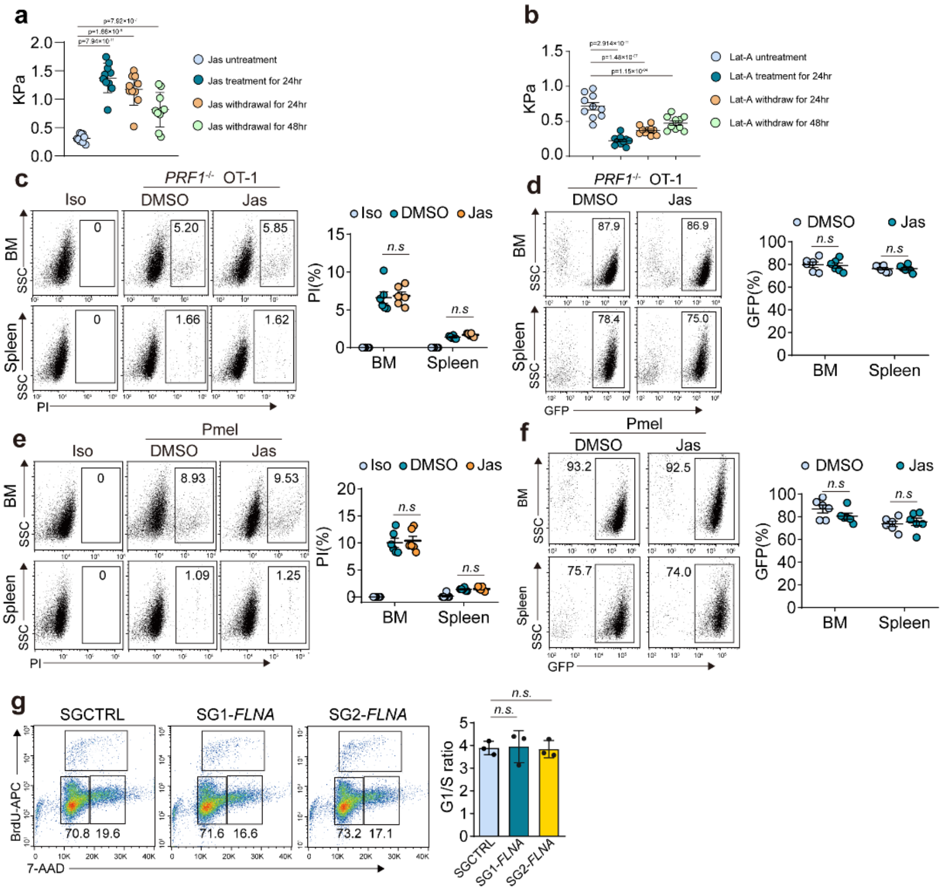

a Molt-4 cells were treated without or with Jas for 24 hours. After Jas treatment or withdrawal, cell stiffness was determined by AFM. **b** YAP-KO Molt-4 cells were treated without or with Lat-A for 24 hours. After Lat-A treatment or withdrawal, cell stiffness was determined by AFM.**c**, **d** NSG mice were transplanted with 2×10^5 OVA-expressing ICN1 leukemic cells. At 7 days post engraftment, NSG mice were treated with or without Jas(50μg/kg), followed by adoptively transferring with 5×10^6 *PRF1*$^{-/-}$ OT-1 T cells. At 10 days post engraftment, PI$^+$ cells in the ICN1 cells were analyzed by flow cytometry (n=6) (**c**). At 15 days post engraftment, leukemia cell dissemination was determined by flow cytometry (n=6) (**d**). **e**, **f** NSG mice were transplanted with 2×10^5 OVA-expressing ICN1 leukemic cells. At 7 days post engraftment, NSG mice were treated with or without Jas(50μg/kg), followed by adoptively transferring with 5×10^6 Pmel T cells. At 10 days post engraftment, PI$^+$ cells in the ICN1 cells were analyzed by flow cytometry (n=6) (**e**). At 15 days post engraftment, leukemia cell dissemination was determined by flow cytometry (n=6) (**f**). **g** Cell growth rate was assessed by flow cytometry using Molt4 cells transfected with SGCTRL or FLNA overexpressed. ICN1, intracellular NOTCH1; BM, bone marrow. NSG, NOD-SCID-Il2rg$^{-/-}$;7-AAD, 7-Aminoactinomycin D. n=10 independent experiments (a and b); n=6 independent experiments (c-f); n=3 independent experiments (g). The data are represented as mean ± SD. *p* value by one-way ANOVA Bonferroni's test (a-g). Source data are provided as a Source Data file.

Supplemental Fig. 7. CTL killing and autolysis is separated by low dose YAP inhibitor

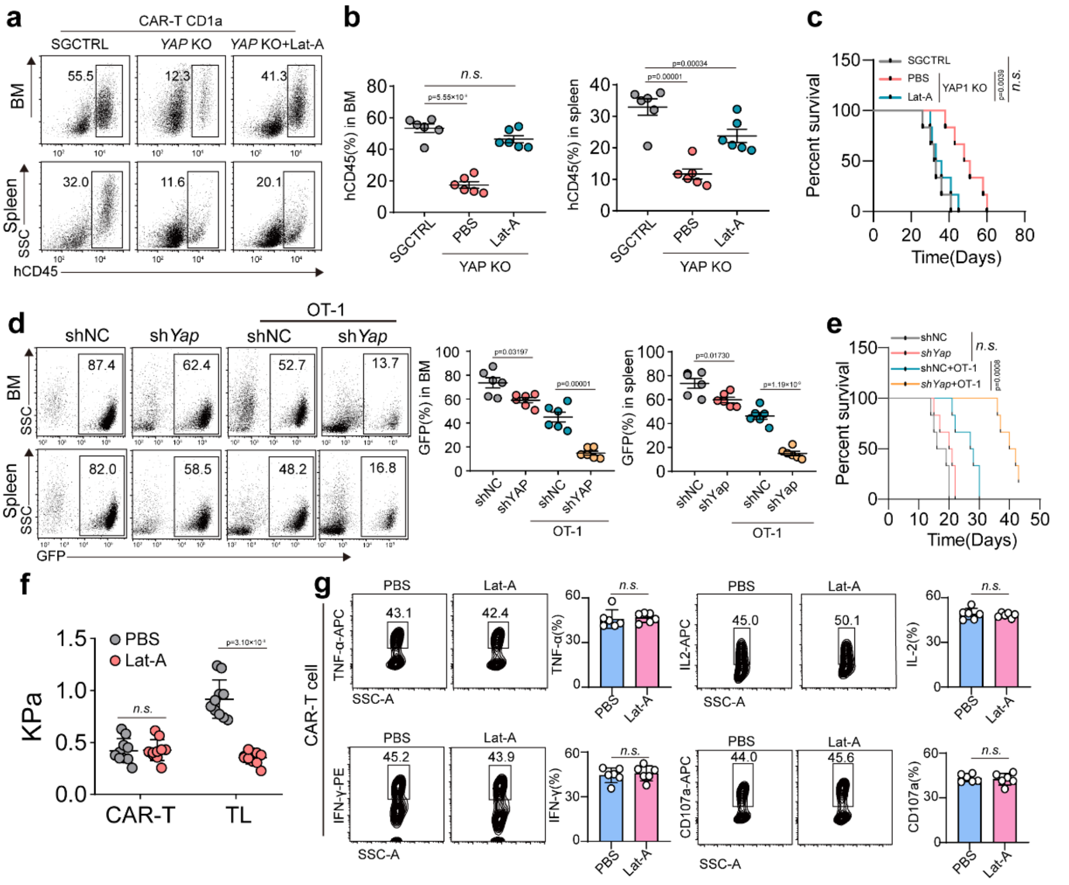

a NSG mice were transplanted with 3×10^{6} YAP knockout and vector control Molt4 cells. At 7 days post engraftment, NSG mice were adoptively transferred with 1×10^{6} human CD1a-CAR-T cells, followed by treating with PBS or latrunculin A (20 ug/kg, intraperitoneally) every 3 d, for a total of 3 times. At 30 days post engraftment, leukemia burden was measured by flow cytometry. **b** The same as **a**, the results from flow cytometry were quantitatively analyzed. **c** the same as **a**, the survival was analyzed. **d**, **e** NSG mice were transplanted with 2×10^{5} OVA-expressing shNC or sh*YAP* ICN cells. At 7 days post engraftment, NSG mice were adoptively transferred with or without 5×10^{6} OT-I T cells. At 15 days post engraftment, leukemia cell dissemination was determined by flow cytometry (**d**). The survival rate was analyzed(**e**). **f, g,** NSG mice were transplanted with 3×10^{6} YAP1 KO Molt4-luc cells. At 7 days post engraftment, NSG mice were adoptively transferred with 1 × 10^{6} human CD1a-CAR, followed by treated with Jas (50µg/kg). 24 hours later, GFP^{+} CAR-T cells and Molt4-luc cells were isolated. The stiffness of CAR-T cells and YAP1 KO Molt4-luc cells was determined by AFM (**f**). CAR-T stimulated with PMA and ionomycin and the expression of TNF-α, IL-2, IFN-γ and CD107a were analyzed by flow cytometry (**g**). n=6 independent experiments (b, d, g); n=10 independent experiments (f). The data are represented as mean ± SD. *p* value by one-way ANOVA Bonferroni's test (b, d, f and g); *p* value by Log-rank survival analysis (c, e). Source data are provided as a Source Data file.

Supplemental Fig. 8. CTL maintain its own softness by MDR1.

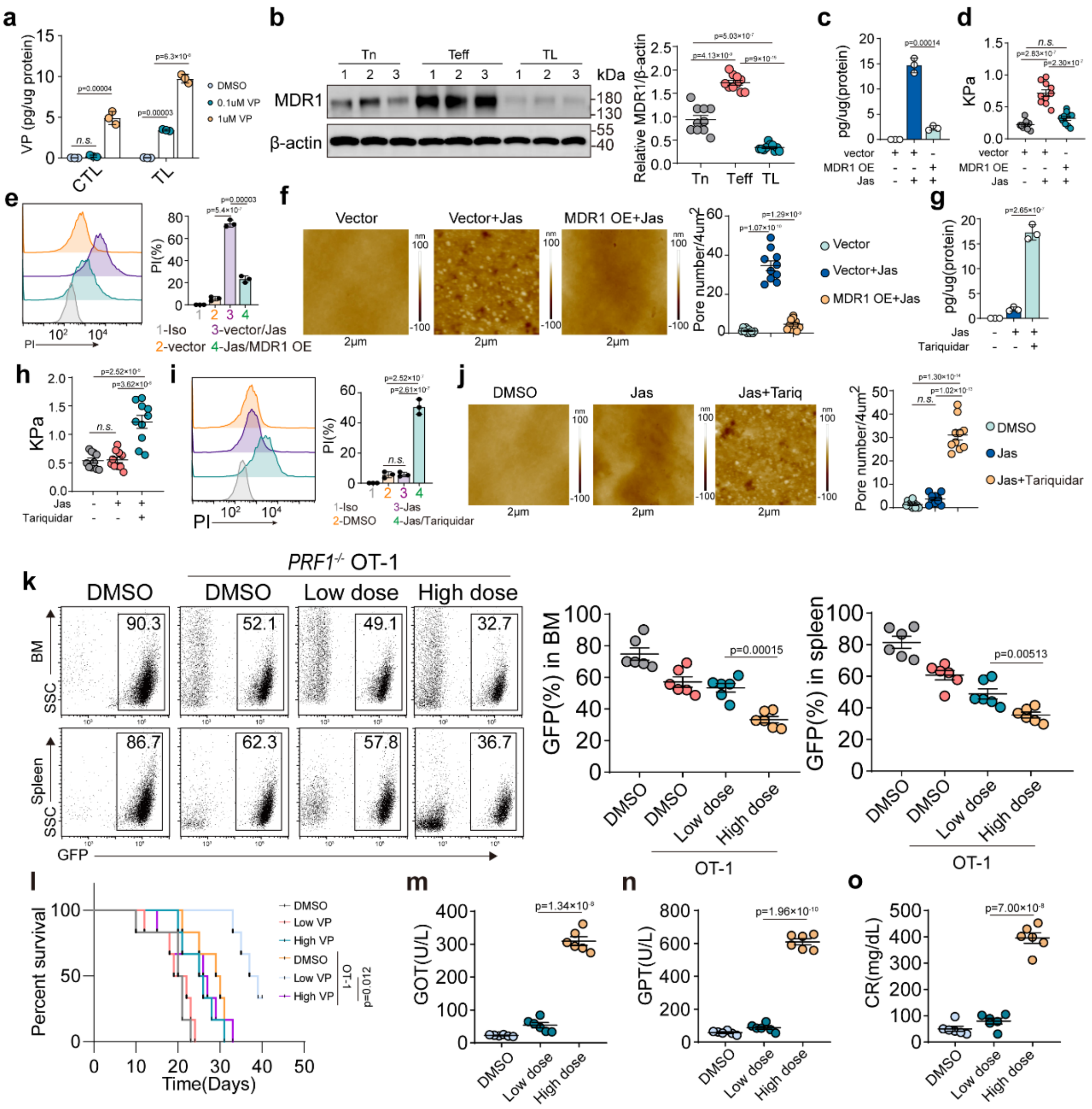

a The levels of verteporfin in CD8+ Teff cells or primary T leukemia cells from patients were analyzed by high performance liquid chromatography (HPLC). **b** The expression of MDR1 in human naïve and effector T cells and primary T leukemia cells were analyzed by western blot. **c, d**, human CD8+ T cells transfected with or without pCMV-MDR1 were treated with 200nM Jas for 12 hr. The levels of Jas in human CD8+ Teff cells were analyzed by high performance liquid chromatography (HPLC) (**c**). The stiffness of cell was analyzed by AFM (**d**). **e, f** the same as (**d**), except that human CD8+ T cells were treated with Perforin for 10 min then were analyzed by flow cytometry (**e**) or AFM (**f**). The formed pore size and number were calculated. **g, h** the same as (**c, d**), except that human CD8+ T cells were treated with 200nM Jas combined with or without 1nM Tariquidar for 12 hr. **i, j** the same as (**e, f**), except that human CD8+ T cells were treated with or without 1nM Tariquidar for 12 hr. **k, l** NSG mice were adoptively transferred with 2×10^5 OVA-expressing ICN1 leukemic cells. At 7 days post engraftment, NSG mice were transferred with 5×10^6 *PRF1*$^{-/-}$ OT-1 T cells, followed by treatment with DMSO or VP (20mg/kg as low dose or 200mg/kg as high dose). At 15 days post engraftment, leukemia cell dissemination was determined by flow cytometry

(**k**). The survival rate was analyzed (**l**). **m-o** The liver and kidney functions were determined by assay kit. n=3 independent experiments (a, c, e, g, i); n=10 independent experiments (b, d, f, h, j); n=6 independent experiments (k, m-o). The data are represented as mean ± SD. *p* value by one-way ANOVA Bonferroni's test (a-k, m-o); *p* value by Log-rank survival analysis (l). Source data are provided as a Source Data file.

Supplemental Fig. 9. Softness promotes T-leukemic cell immune evasion in patients

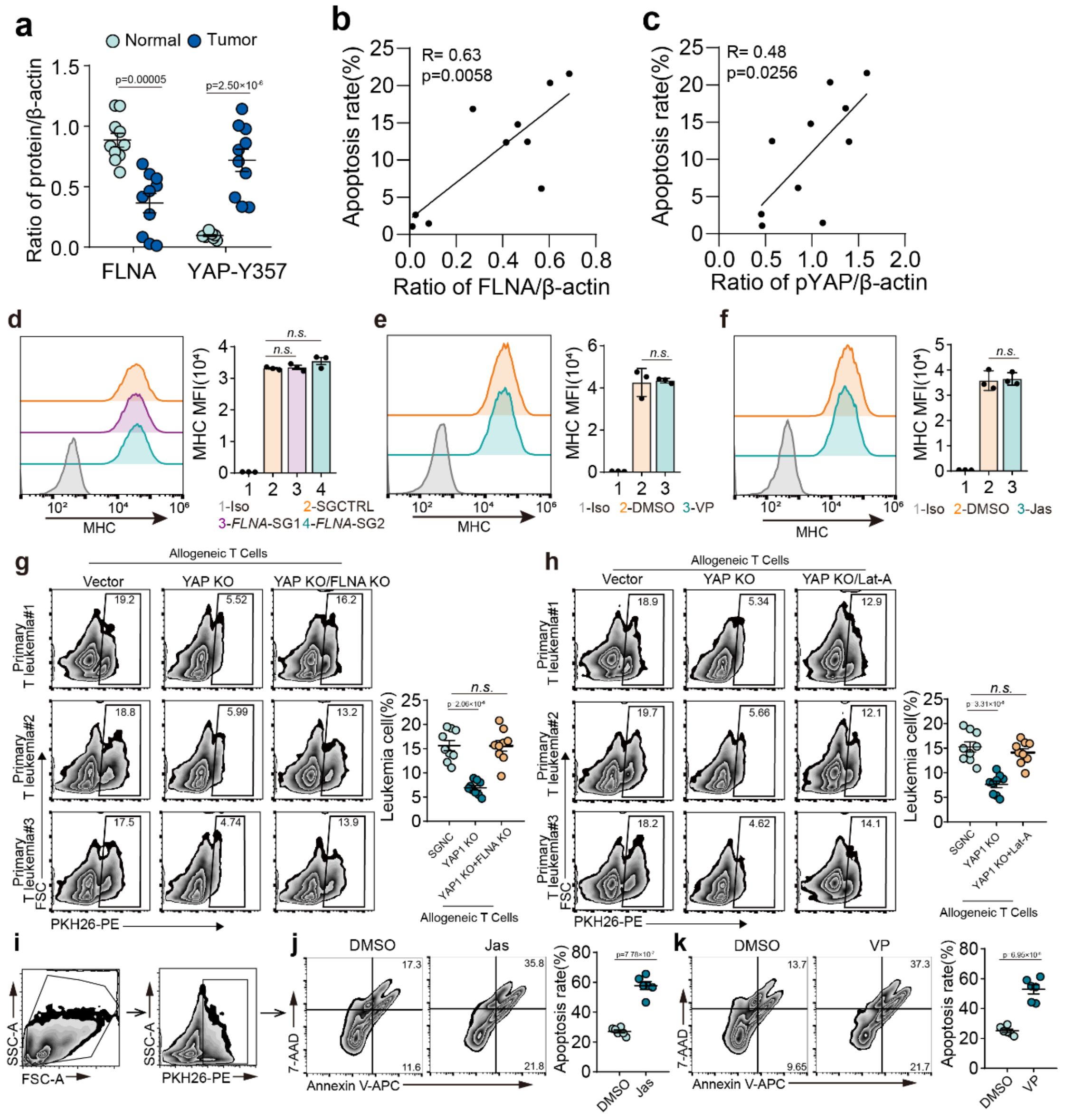

a The expression of FLNA and Y357-YAP in normal human T cells and primary T leukemia cells were analyzed by western blot. Western blot data were quantitatively analyzed by Image J. **b, c** Cell viability of the primary T leukemia cells, primary B-leukemic and acute myeloid leukemic cells were detected by flow cytometry after coculturing with allogeneic T cells for 12 hr. The correlation between FLNA (**b**) and phosphorylated YAP1 (Y357) (**c**) and apoptosis rate was showed. **d** The expression of MHC-I in SGCTRL or SGFLNA-Molt4 cells was analyzed by flow cytometry. **e, f** The expression of MHC-I in Molt4 cells was analyzed by flow cytometry after VP (**e**) or Jas (**f**) treatment for 24 hr. **g, h** NSG mice were irradiated (2 Gy), then adoptively transferred with 1×10^6 PKH26-labeled primary leukemic T cells transfected with vector, YAP1 KO or YAP1/FLNA dual KO and 1×10^6 allogeneic T cells. One day later, NSG mice were treated with PBS or Lat-A (20 ug/kg). On day 3, $PKH26^+$ cells were analyzed

by flow cytometry (g). Representative flow plots and quantification of percent of $PKH26^+$ primary T leukemia cells in bone marrow from mice (h). **i-k** NSG mice were irradiated (2 Gy), then adoptively transferred with 1×10^6 primary T leukemic cells and 1×10^6 allogeneic T cells (**i**). At 1 day post engraftment, NSG mice were treated with Jas (50μg/kg) (**j**) or VP (20mg/kg) (**k**). At 3 days post engraftment, the cell viability of primary T leukemic cells in bone marrow from mice treated with Jas or VP was detected by flow cytometry. n=10 independent experiments (a, g, h); n=3 independent experiments (d-f); n=6 independent experiments (j, k). The data are represented as mean ± SD. *p* value by one-way ANOVA Bonferroni's test (a, d-h); *p* value by two-tailed Student's t-test (j-k). Source data are provided as a Source Data file.

Supplemental Fig. 10. Cytoskeletal agents had no effect on effector T cells but did alter T leukemic cells

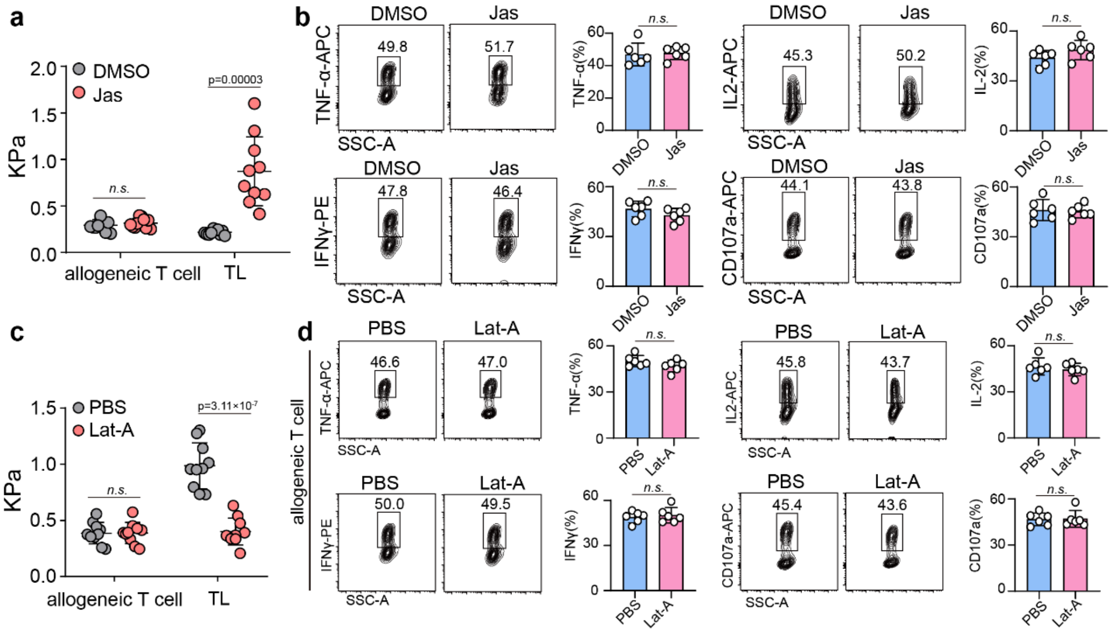

a, b NSG mice were irradiated (2 Gy), then adoptively transferred with 1×10^6 primary leukemic T cells labeled with PHK26 and 1×10^6 allogeneic T cells labeled with CFSE. At 1 day post engraftment, NSG mice were treated with Jas (50μg/kg). At 3 days post engraftment, $CFSE^+$ allogeneic T cells were isolated and stimulated with PMA and ionomycin. The stiffness of allogeneic T cells and primary leukemic T cells was determined by AFM (**a**). The expression of TNF-α, IL-2, IFN-γ and CD107a were analyzed by flow cytometry (n=6) (**b**). **c, d** the same as (**a, b**), except that NSG mice were treated with Lat-A (20μg/kg). n=10 independent experiments (a, c); n=6 independent experiments (b, d). The data are represented as mean ± SD. *p* value by one-way ANOVA Bonferroni's test (a, c); *p* value by two-tailed Student's t-test (b, d). Source data are provided as a Source Data file.

Supplemental Fig. 11 Gating strategies used in FACS analysis.

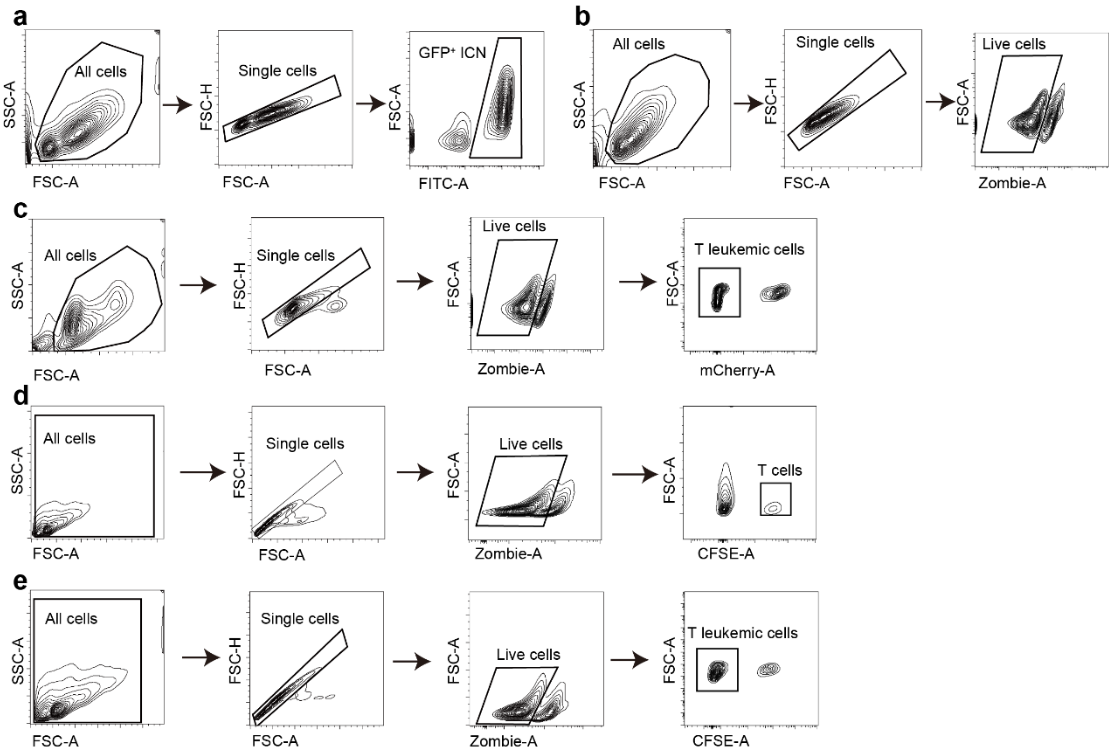

a Gating strategy to analyse GFP$^+$ ICN cells analysis presented on Fig.6b and Supplemental Fig.6b, 6d. **b** Gating strategy to analyse single cells analysis presented on Fig.6e, 6f, 8k, 8l and Supplemental Fig.6c, 6e, 7d, 8k. **c** Gating strategy to analyze mCherry$^-$ T leukemic cells analysis presented on Fig.7d, 7e, 8a, 8b and Supplemental Fig.7a, 7b. **d** Gating strategy to analyse CFSE stained activation CD8$^+$ T cells analysis presented on Supplemental Fig.7g, 10b, 10d. **e** Gating strategy to analyse T leukemic cells presented on Fig.9e-i and Supplemental Fig.9g, 9h.

Supplementary Table 1. Clinical information of patients

Patients NO.	Gender	Age	Status	Sample
1	Male	49	Newly diagnosis	T leukemia
2	Male	27	Newly diagnosis	T leukemia
3	Female	61	Newly diagnosis	T leukemia
4	Female	36	Newly diagnosis	T leukemia
5	Male	53	Newly diagnosis	T leukemia
6	Male	32	Newly diagnosis	T leukemia
7	Male	51	Newly diagnosis	T leukemia
8	Male	17	Newly diagnosis	T leukemia
9	Female	28	Newly diagnosis	T leukemia
10	Male	41	Newly diagnosis	T leukemia
11	Male	79	Newly diagnosis	T leukemia
12	Female	59	Newly diagnosis	T leukemia
13	Female	45	Newly diagnosis	T leukemia
14	Male	72	Newly diagnosis	T leukemia
15	Female	48	Newly diagnosis	T leukemia
16	Male	29	Newly diagnosis	T leukemia
17	Male	17	Newly diagnosis	T leukemia
18	Female	77	Newly diagnosis	T leukemia
19	Female	49	Newly diagnosis	T leukemia
20	Male	51	Newly diagnosis	T leukemia
21	Male	20	Newly diagnosis	B-ALL
22	Female	36	Newly diagnosis	B-ALL
23	Female	19	Newly diagnosis	B-ALL
24	Male	33	Newly diagnosis	B-ALL
25	Female	21	Newly diagnosis	B-ALL
26	Male	45	Newly diagnosis	B-ALL
27	Male	42	Newly diagnosis	AML
28	Female	50	Newly diagnosis	AML
29	Female	62	Newly diagnosis	AML
30	Male	55	Newly diagnosis	AML
31	Female	63	Newly diagnosis	AML
32	Male	44	Newly diagnosis	AML

Supplementary Table 2. Sequence of shRNA and pcr primer.

Name	Forward	Reverse
**Primer sequences for qRT-PCR**		
β-ACTIN	5'-CATGTACGTTGCTATCCAGGC-3'	5'-CTCCTTAATGTCACGCACGAT-3'
FLNA	5'-CTTATCGCGCTGTTGGAGGT-3'	5'-GCCACCGACACGTTCTCAA-3'
YAP1	5'-TAGCCCTGCGTAGCCAGTTA-3'	5'-TCATGCTTAGTCCACTGTCTGT-3'
TEAD1	5'-ATGGAAAGGATGAGTGACTCTGC-3'	5'-TCCCACATGGTGGATAGATAGC-3'
β-actin	5'-GGCTGTATTCCCCTCCA TCG-3'	5'-CCAGTTGGTAACAATGCCATGT-3'
flna	5'-TCCCCAACCGGGCAAATATG-3'	5'-CTGGCTACCCTGAGGATAGTT-3'
yap1	5'-TGAGATCCCTGATGATGTACCAC-3'	5'-TGTTGTTGTCTGATCGTTGTGAT-3'
tead1	5'-GAGCGACTCGGCAGATAAGC-3'	5'-CCACACGGCGGATAGATAGC-3'
**Primer sequences for siRNA**		
yap-1	CCACCAAGCTAGATAAAGA	
yap-2	GGAGAAGTTTACTACATAA	
FLNA-1	CCCGCCTGTCACTGCAGCTGC	
FLNA-2	CCCACCCACTTCACAGTAAAT	
TEAD1-1	CCACTGCCATTCATAACAA	
TEAD1-2	GTACGAGAGTTCTGAAAAT	
ZAP70-1	CAGGCGTAGATCACCAGAATA	
ZAP70-2	GAAGCCCTACAAGAAGATGAA	
LCK-1	GCACACATCAGGAGTTCAATA	
LCK-2	AGCCATTAACTACGGGACATT	
**Primer sequences for sgRNA**		
SGCTRL (human)	5'-CACCGGGGCGAGGAGCTGTTCACCG-3'	5'-AAACCGGTGAACAGCTCCTCGCCCC-3'
YAP-SGRNA1 (human)	5'-TCGAACATGCTGTGGAGTCA -3'	5'-TGACTCCACAGCATGTTCGA-3'
YAP-SGRNA2(human)	5'-CAACTGCAGAGAAGCTGGAG-3'	5'-CTCCAGCTTCTCTGCAGTTG-3'
FLNA-CRISPa-1(human)	5'-GACCCCGGAGGTAAGAGGCAC-3'	5'-GTGCCTCTTACCTCCGGGGTC-3'
FLNA-CRISPa-2(human)	5'-GCTGGTCAGGGCCTTCACGA-3'	5'-TCGTGAAGGCCCTGACCAGC-3'
**Primer sequences for CHIP qPCR**		
FLNA	5'-CAGCCTCAGAGAGTTTCCCG-3'	5'-AACGTTAACAGCTCAGTGAACA-3'
flna	5'-AGGACAGTCATTTTCATGAAC-3'	5'-TGCAGAGTCAATTTGGTGATC-3'
**Primer sequences for shRNA**		
scramble for mouse	CCGGGTGGATGCAACTGCGTATCATCTCGAGATGATACGCAGTTGCATCCACTTTTTG	AATTCAAAAAGTGGATGCAACTGCGTATCATCTCGAGATGATACGCAGTTGCATCCAC
Yap1-shRNA1(mouse)	CGGGCGGTTGAAACAACAGGAATTATCGATAATTCCTGTTGTTTCAACCGTTTTTG	AATTCAAAAACGGTTGAAACAACAGGAATTATCGATAATTCCTGTTGTTTCAACCG
Yap1-shRNA2(mouse)	CCGGCCACCAAGCTAGATAAAGAAATCGATTTCTTTATCTAGCTTGGTGGTTTTTG	AATTCAAAAACCACCAAGCTAGATAAAGAAATCGATTTCTTTATCTAGCTTGGTGG

Supplementary Table 3. Primer sequences for qRT-PCR, siRNA, and sgRNA assays							
Antibodies used for Western blotting (WB) and immunoprecipitation (IP).							
Primary antibodies	Supplier	Catalogue	Application	Host species	Species activity	clone	Dilution
anti-FLNA	Cell Signaling Technology	4762	WB	Rabbit	Hu, Mo	NA	1:1000
anti-β-actin	Cell Signaling Technology	3700	WB	Mouse	Hu, Mo	8H10D10	1:1000
anti-YAP	Cell Signaling Technology	4912	WB, IP	Rabbit	Hu, Mo	NA	1:1000
anti-YAP(phospho Y357)	abcam	ab62751	WB, IF	Rabbit	Hu	NA	1:1000
anti-p-YAP	Cell Signaling Technology	13008	WB, IP	Rabbit	Hu, Mo	D9W2I	1:1000
anti-Tead1	GeneTex	GTX32918	WB, IF, IP	Rabbit	Hu, Mo	NA	1:1000
anti-ZAP70	Cell Signaling Technology	3165S	WB, IF, IP	Rabbit	Hu, Mo	D1C10E	1:1000
anti-pZAP70	Cell Signaling Technology	2704s	WB, IP	Rabbit	Hu	NA	1:1000
anti-LCK	Cell Signaling Technology	2984s	WB	Rabbit	Hu	D88	1:1000
anti-p-LCK	abcam	ab138442	WB	Rabbit	Hu	NA	1:1000
anti-MDR1	Cell Signaling Technology	13978s	WB	Rabbit	Hu, Mo	E1Y7S	1:1000
anti-flag	Cell Signaling Technology	8146	WB, IP, IF	Mouse	Hu, Mo	9A3	1:1000
anti-Histone H3	Cell Signaling Technology	12648	WB	Rabbit	Hu, Mo	D1H2	1:1000
Antibodies used for flow cytometric analysis							
Primary antibodies	Supplier	Catalogue	Application	Host species	Species activity	clone	Dilutio
APC anti-mouse CD45	Biolegend	147708	Fc	Rat	Mo	I3/2.3	0.5ug per test
FITC anti-human CD45	Biolegend	304006	Fc	mouse	Hu	HI30	0.5ug per test
APC anti-mouse IFN-γ	Biolegend	505810	Fc	Rat	Mo	XMG1.2	0.5ug per test
PE anti-mouse TNF-α	Biolegend	506306	Fc	Rat	Mo	MP6-XT22	0.5ug per test
APC anti-BrdU	Biolegend	364114	Fc	mouse	Mo	3D4	1ul per test
Antibodies used for immunofluorescence (IF)							
Primary antibodies	Supplier	Catalogue	Application	Host species	Species activity	clone	Dilutio
anti-YAP	Abcam	ab52771	WB, IP, IF	Rabbit	Hu	EP1674Y	1:200
anti-FLNA	Abcam	76289	WB, IF	Rabbit	Hu, Mo	EP2405Y	1:200
Phalloidin-iFluor	Abcam	176753	IF	NA	NA	NA	1:1000
