# Supplementary material for: Cell softness renders cytotoxic T lymphocytes and T leukemic cells resistant to perforin-mediated killing
Source: Nat Commun. 2024 Feb 15;15:1405. doi: 10.1038/s41467-024-45750-w (PMC10869718; doi:10.1038/s41467-024-45750-w)
Supplement: Supplementary file 3 — Reporting Summary [file 41467_2024_45750_MOESM3_ESM.pdf]

nature portfolio

Corresponding author(s): Bo Huang and Xiaohui Zhang

Last updated by author(s): Dec 21, 2023

Reporting Summary

Nature Portfolio wishes to improve the reproducibility of the work that we publish. This form provides structure for consistency and transparency in reporting. For further information on Nature Portfolio policies, see our Editorial Policies and the Editorial Policy Checklist.

Statistics

For all statistical analyses, confirm that the following items are present in the figure legend, table legend, main text, or Methods section.

n/a	Confirmed	
☐	☒	The exact sample size (*n*) for each experimental group/condition, given as a discrete number and unit of measurement
☐	☒	A statement on whether measurements were taken from distinct samples or whether the same sample was measured repeatedly
☐	☒	The statistical test(s) used AND whether they are one- or two-sided<br>*Only common tests should be described solely by name; describe more complex techniques in the Methods section.*
☒	☐	A description of all covariates tested
☒	☐	A description of any assumptions or corrections, such as tests of normality and adjustment for multiple comparisons
☐	☒	A full description of the statistical parameters including central tendency (e.g. means) or other basic estimates (e.g. regression coefficient) AND variation (e.g. standard deviation) or associated estimates of uncertainty (e.g. confidence intervals)
☐	☒	For null hypothesis testing, the test statistic (e.g. *F*, *t*, *r*) with confidence intervals, effect sizes, degrees of freedom and *P* value noted<br>*Give P values as exact values whenever suitable.*
☒	☐	For Bayesian analysis, information on the choice of priors and Markov chain Monte Carlo settings
☒	☐	For hierarchical and complex designs, identification of the appropriate level for tests and full reporting of outcomes
☐	☒	Estimates of effect sizes (e.g. Cohen's *d*, Pearson's *r*), indicating how they were calculated

Our web collection on statistics for biologists contains articles on many of the points above.

Software and code

Policy information about availability of computer code

Data collection	Nikon Instruments A1 Confocal Laser Microscope(Nikon Instruments A1 Confocal Laser Microscope Series With NIS-Elements C Software),<br>BD Accuri™ C6 Plus(C6 Plus Analysis Software for PC),<br>ABI QuantStudio 3 RT PCR 96-well (QuantStudio 3 and 5 Real-Time PCR System Software),<br>The Dimension Icon AFM (NanoScope Analysis v1.40r1)<br>MAGeCK (0.5.9),<br>ÄKTA pure 25 M(Cytiva)<br>Living Imaging IVIS® Spectrum In Vivo Imaging System
Data analysis	GraphPad Prism 8.0.0 was used for analysis of in vivo and in vitro phenotypic assays and for producing graphs. ImageJ v. 1.52 was used for the visualization and presentation of western blots, and analysis of immunohistochemical staining. QuantStudio Design & Analysis Software 1.5 was used to analyse the qPCR results. MAGeCK (0.5.9), was used for the CRISPR screening data. NanoScope Analysis 1.9 was used for the visualization and presentation of the shape of cell surfaces , and analysis of the pore number. JPK Data processing was used for analysis of the softness of cells.

For manuscripts utilizing custom algorithms or software that are central to the research but not yet described in published literature, software must be made available to editors and reviewers. We strongly encourage code deposition in a community repository (e.g. GitHub). See the Nature Portfolio guidelines for submitting code & software for further information.

Data

Policy information about availability of data

All manuscripts must include a data availability statement. This statement should provide the following information, where applicable:

- Accession codes, unique identifiers, or web links for publicly available datasets
- A description of any restrictions on data availability
- For clinical datasets or third party data, please ensure that the statement adheres to our policy

The CRISPR screening data generated in this study have been deposited in the Science Data Bank database [https://www.scidb.cn/en/s/JfqUZb]. Publicly available datasets in NCBI were used for Figure 4 found at accession numbers: GSE26713. All the other data supporting the findings of this study are available within the article and its Supplementary Information files. Source data are provided with this paper.

Research involving human participants, their data, or biological material

Policy information about studies with human participants or human data. See also policy information about sex, gender (identity/presentation), and sexual orientation and race, ethnicity and racism.

Reporting on sex and gender	We have fully considered the factor of sex in our experimental design. Our study includes 18 male and 14 female participants.
Reporting on race, ethnicity, or other socially relevant groupings	The findings in this study were not involved in race, ethnicity, or other socially relevant groupings.
Population characteristics	The patient experiments have obtained approval from the Ethics Committee of Peking University People's Hospital. Relevant patient information has been included in Supplementary Table 1.
Recruitment	All samples were obtained from the Peking University People's Hospital. All patients were diagnosed with leukemia and collected with informed consent.
Ethics oversight	All donors provided informed written consent before sampling according to the Declaration of Helsinki, and the present study was approved by the institutional ethics committees of Committee of Peking University People's Hospital (NKRDP2021005-EC-2).

Note that full information on the approval of the study protocol must also be provided in the manuscript.

Field-specific reporting

Please select the one below that is the best fit for your research. If you are not sure, read the appropriate sections before making your selection.

☒ Life sciences ☐ Behavioural & social sciences ☐ Ecological, evolutionary & environmental sciences

For a reference copy of the document with all sections, see nature.com/documents/nr-reporting-summary-flat.pdf

Life sciences study design

All studies must disclose on these points even when the disclosure is negative.

Sample size	Sample sizes were determined on the basis of estimates from preliminary experiments.
Data exclusions	No data were excluded from the analysis.
Replication	At least three biologically independent experiments were performed in each case, unless otherwise stated in the respective figure legend. Replicates were reproducible.
Randomization	For the animal study, mice were randomly divided into different groups to investigated leukemia growth and mouse survival. The tests were also randomly selected from all samples.
Blinding	All in vivo experiments were performed blindly.<br>For in vitro experiments, researchers had information about the group by necessity to prepare RNA and protein samples from each groups. However, individuals performing analysis with these samples were blinded to the group allocation.

Reporting for specific materials, systems and methods

We require information from authors about some types of materials, experimental systems and methods used in many studies. Here, indicate whether each material, system or method listed is relevant to your study. If you are not sure if a list item applies to your research, read the appropriate section before selecting a response.

Materials & experimental systems

n/a	Involved in the study
☐	☒ Antibodies
☐	☒ Eukaryotic cell lines
☒	☐ Palaeontology and archaeology
☐	☒ Animals and other organisms
☒	☐ Clinical data
☒	☐ Dual use research of concern
☒	☐ Plants

Methods

n/a	Involved in the study
☒	☐ ChIP-seq
☐	☒ Flow cytometry
☒	☐ MRI-based neuroimaging

Antibodies

Antibodies used	Western blot: anti-β-actin (Cell Signaling Technology, Cat.: 3700; 1:1000), anti-FLNA (Cell Signaling Technology, Cat.: 4762; 1:1000), anti-YAP1 (Cell Signaling Technology, Cat.: 4912; 1:1000), anti-p-YAP1(Ser127) (Cell Signaling Technology, Cat.: 13008; 1:1000), anti-YAP1 (phospho Y357) (abcam, Cat.: ab62751; 1:1000), anti-flag (Cell Signaling Technology, Cat.: 8146; 1:1000), anti-Histone H3 (Cell Signaling Technology, Cat.: 12648; 1:1000), anti-ZAP-70 (Cell Signaling Technology, Cat.: 3165S; 1:1000), anti-p-ZAP-70(Try493) (Cell Signaling Technology, Cat.: 2704s; 1:1000), anti-LCK (Cell Signaling Technology, Cat.: 2984s; 1:1000), anti-Lck (phospho Y393)(abcam, Cat.: ab138442;1:1000), anti-MDR1 (Cell Signaling Technology, Cat.: 13978s; 1:1000), anti-Tead1 (GeneTex, Cat.: GTX32918; 1:1000). immunofluorescence:anti-YAP (Abcam,Cat.: ab52771; 1:200),anti-FLNA (Abcam, Cat.: ab76289; 1:200)
Validation	All antibodies were purchased from the vendors mentioned above. Antibodies used for Western blot were validated by their manufacture companies. These antibodies are routinely used in our laboratory.

Eukaryotic cell lines

Policy information about cell lines and Sex and Gender in Research

Cell line source(s)	Mouse tumor cell lines B16, OVA-B16 (melanoma) and human tumor cell lines MCF7 (breast cancer) were purchased from China Center for Type Culture Collection (Beijing, China).The 293T, Jurkat, and MOLT4 cells were purchased from American Type Culture Collection (ATCC,USA)
Authentication	None of the cell lines were independently authenticated.
Mycoplasma contamination	Our cell lines are routinely tested for mycoplasma. None of the cell lines used in this study have tested positive for mycoplasma.
Commonly misidentified lines (See ICLAC register)	No commonly misidentified cell lines were used.

Animals and other research organisms

Policy information about studies involving animals; ARRIVE guidelines recommended for reporting animal research, and Sex and Gender in Research

Laboratory animals	All mice, under a specific pathogen-free environment, were housed in a temperature-controlled environment of 22–23°C, and 40–70% humidity in individually ventilated cages with wood pieces as bedding with 12h light/dark cycles and received food and water ad libitum (Jiangsu Xietong Pharmaceutical Bio-engineering, cat.1010001, Jiangsu). For all in vivo experiments, mice allocated to different experimental groups were sex-, age- and housing-matched. Each group of mice were cohoused separately.Six- to Eight-week-old male or female C57BL/6J Nifdc (stock number: 000664) or NOD-SCID-Il2rg−/−(NSG) mice (stock number: 005557) were purchased from the Center of Medical Experimental Animals of Chinese Academy of Medical Science (Beijing, China). Pmel-1 transgenic (B6.Cg-Thy1a/Cy Tg(TcraTcrb)8Rest/J) mice were presented by Dr. Ying Wan (Third Military Medical University, China). PRF1-/- (C57BL/6-Prf1tm1Sdz/J, referred to as PRF1−/−,) mice were obtained from Shanghai Model Organisms Center, Inc (Shanghai, China). OT-I transgenic (C57BL/6-Tg (TcraTcrb)1100Mjb/J) mice were gifted by Dr. Hui Zhang (Sun Yat-Sen University, China) and were crossed to PRF1−/− mice. ICN mouse model was gifted by Dr. Tao Cheng (State Key Laboratory of Experimental Hematology, Institute of Hematology and Blood Diseases Hospital).
Wild animals	No Wild animals were used in this study.
Reporting on sex	The findings in this study used both male and female samples.
Field-collected samples	No Field-collected samples were used in this study.
Ethics oversight	These animals were maintained in the Animal Facilities of the Chinese Academy of Medical Science under pathogen-free conditions. All studies involving mice were approved by the Animal Care and Use Committee of the Chinese Academy of Medical Science (ACUC-A02-2022-085).

Note that full information on the approval of the study protocol must also be provided in the manuscript.

Flow Cytometry

Plots

Confirm that:

☒ The axis labels state the marker and fluorochrome used (e.g. CD4-FITC).

☒ The axis scales are clearly visible. Include numbers along axes only for bottom left plot of group (a 'group' is an analysis of identical markers).

☒ All plots are contour plots with outliers or pseudocolor plots.

☒ A numerical value for number of cells or percentage (with statistics) is provided.

Methodology

Sample preparation	Immune cells or tumor cells from various experiments were stained with APC anti-mouse CD45 (Cat: 147708; Biolegend, USA) and FITC anti-mouse CD45 (Cat: 304006; Biolegend, USA). Intracellular staining APC anti-mouse IFN-γ (Cat: 505810; Biolegend, USA) and PE anti-mouse TNF-α (Cat: 506306; Biolegend, USA) was carried out using Intracellular Fixation & Permeabilization Buffer Set (Invitrogen) according to the manufacturer's instructions. APC Annexin V(Cat:640919; Biolegend, USA) and propidium iodide(Cat:537059;Sigma) used to detect apoptosis.For PI staining, cells in HBSS solution with 2% BSA and 2mM CaCl2 were treated with perforin for the indicated time, and then performed the flow cytometry analysis. Data was analyzed with Flowjo software.
Instrument	Accuri C6 (BD Biosciences) and Invitrogen Attune NxT (Themro)
Software	FlowJo software.
Cell population abundance	The fluorescence intensity of human or mouse CD8+ T and T leukemia cells was analyzed.
Gating strategy	All gates were set based on FMO (full minus one) stains and isotype control antibodies after appropriate compensation using single-stained compensation controls.

☒ Tick this box to confirm that a figure exemplifying the gating strategy is provided in the Supplementary Information.
